# Transcriptome sequencing and bioinformatics analysis of gastrocnemius muscle in type 2 diabetes mellitus rats

**DOI:** 10.1186/s12891-024-07568-x

**Published:** 2024-06-08

**Authors:** Kuishuai Xu, Liang Zhang, Tianrui Wang, Tengbo Yu, Xia Zhao, Yingze Zhang

**Affiliations:** 1 Department of Sports Medicine, The Affiliated Hospital of Qingdao University, Qingdao, Shandong 266000 China; 2 Department of Abdominal ultrasound, The Affiliated Hospital of Qingdao University, Qingdao, Shandong 266000 China; 3 Department of Traumatology, The Affiliated Hospital of Qingdao University, Qingdao, Shandong 266000 China; 4 Department of Orthopedic Surgery, Qingdao Municipal Hospital, Qingdao, Shandong 266000 China

**Keywords:** Type 2 diabetes mellitus, Gastrocnemius muscle, Sarcopenia, Transcriptomics, Apoptosis

## Abstract

**Objective:**

Type 2 diabetes mellitus (T2DM) is one of the high risk factors for sarcopenia. However, the pathogenesis of diabetic sarcopenia has not been fully elucidated. This study obtained transcriptome profiles of gastrocnemius muscle in normal and T2DM rats based on high-throughput sequencing technology, which may provide new ideas for exploring the pathogenesis of diabetic sarcopenia.

**Methods:**

Twelve adult male Sprague-Dawley rats were randomly divided into Control group and T2DM group, and gastrocnemius muscle tissue was retained for transcriptome sequencing and real-time quantitative polymerase chain reaction (qRT-PCR) 6 months later. Screening differentially expressed genes (DEGs), Cluster analysis, gene ontology (GO) functional annotation analysis and Kyoto Encyclopedia of Genes and Gnomes (KEGG) functional annotation and enrichment analysis were performed for DEGs. Six DEGs related to apoptosis were selected for qTR-PCR verification.

**Results:**

Transcriptomic analysis showed that there were 1016 DEGs between the gastrocnemius muscle of T2DM and normal rats, among which 665 DEGs were up-regulated and 351 DEGs were down-regulated. GO analysis showed that the extracellular matrix organization was the most enriched in biological processes, with 26 DEGs. The extracellular matrix with 35 DEGs was the most abundant cellular component. The extracellular matrix structural constituent, with 26 DEGs, was the most enriched in molecular functions. The highest number of DEGs enriched in biological processes, cellular components and molecular functions were positive regulation of transcription by RNA polymerase II, nucleus and metal ion binding, respectively. There were 78, 230 and 89 DEGs respectively. KEGG pathway enrichment analysis showed that ECM-receptor interaction, PI3K-Akt signaling pathway and TGF-β signaling pathway(*p* < 0.001) had higher enrichment degree and number of DEGs. qRT-PCR results showed that the fold change of Map3k14, Atf4, Pik3r1, Il3ra, Gadd45b and Bid were 1.95, 3.25, 2.97, 2.38, 0.43 and 3.6, respectively. The fold change of transcriptome sequencing were 3.45, 2.21, 2.59, 5.39, 0.49 and 2.78, respectively. The transcriptional trends obtained by qRT-PCR were consistent with those obtained by transcriptome sequencing.

**Conclusions:**

Transcriptomic analysis was used to obtain the “gene profiles” of gastrocnemius muscle of T2DM and normal rats. qRT-PCR verification showed that the genes related to apoptosis were differentially expressed. These DEGs and enrichment pathways may provide new ideas for exploring the pathogenesis of diabetic sarcopenia.

**Supplementary Information:**

The online version contains supplementary material available at 10.1186/s12891-024-07568-x.

## Introduction

Diabetes is a metabolic disease characterized by hyperglycemia, and the number of diabetic patients has increased dramatically in recent years [1]. In addition to the common macrovascular and microvascular complications, people with diabetes also have decreased muscle strength and function. This age-related loss of muscle mass, combined with decreased muscle strength and physical function, is known as sarcopenia [2]. Sarcopenia has now been included in the International Classification of Diseases as an independent disease. In the process of the rapid development of global population aging, sarcopenia has become a mainstream public health problem [3]. At the same time, more and more studies have begun to pay attention to individuals with diabetic sarcopenia. Studies have shown that the occurrence of diabetic sarcopenia will increase the risk of falls and disabilities, reduce the quality of life, and increase the mortality of patients [4]. Recent studies have shown that T2DM is closely related to sarcopenia. With the progression of T2DM, the skeletal muscle function and muscle mass of patients will gradually decrease, and the prevalence of sarcopenia is much higher than that of the normal population. Wang et al. [5] showed that the prevalence of sarcopenia in T2DM patients was 1.56 times that of healthy people. The study of Kim et al. [6] showed that patients with diabetes had a 3 times higher risk of sarcopenia than patients without diabetes. Sarcopenia has received increasing attention due to its severe impact on the quality of life in elderly T2DM patients, and has therefore been recognized as a third category of disabling complications in T2DM patients in addition to microvascular and macrovascular complications. At present, the exact mechanism of diabetic tendinopathy has not been fully elucidated, and there is a lack of targeted interventions and drugs. Therefore, the in-depth study of the pathological mechanism of diabetic sarcopenia and the search for new therapeutic targets have important guiding significance for the clinical prevention and control of diabetic sarcopenia.

With the development of systems biology, omics technology, which consists of transcriptomics, proteomics, genomics and metabolomics, has been widely used in basic research. Transcriptomics is a discipline that studies the transcriptional regulation of all genes in cells at the overall level. It has become an important bridge between genomic genetic information and biological functions, and is of great significance in interpreting the functional composition of genomes and revealing the mechanisms of body development and diseases [7]. The first transcriptome study of muscle cells from 5 participants with strength training and 5 controls with sedentary lifestyles identified 69 differentially expressed genes [7]. Another important finding achieved by RNA sequencing is that skeletal muscle dysregulation during bed rest in the old may be driven by alterations in molecules related to fibrosis, inflammation, and cell adhesion [8]. The role of altered mitochondrial function in the pathological loss of skeletal muscle mass and function in older people was also reported [9].Although transcriptomics has been applied in the research field of sarcopenia, as one of the complications of diabetes, the report of transcriptomics on diabetic sarcopenia has not been published.

Although the exact mechanisms underlying sarcopenia are far to be unveiled, accumulating preclinical evidence suggests that an age-related acceleration of myocytes loss via apoptosis might represent a key mechanism driving the onset and progression of muscle loss [10–12]. Based on this, transcriptome sequencing was performed on gastrocnemius muscle of T2DM and normal rats in this study to screen out differential genes that may be involved in regulating apoptosis, and qPT-PCR was performed on them. This study further explores the potential genes that play a regulatory role in the process of sarcopenia induced by T2DM, and provides a reference for elucidation of the molecular mechanism of diabetic sarcopenia.

## Materials and methods

### Animal model and grouping

Twelve 8-week-old Sprague-Dawley rats were provided by Animal Center of Qingdao University and randomly divided into Control group and T2DM group, with 6 rats in each group. All animals were raised in clean, suitable (20–24 ° C) animal houses, and were accommodated 2 weeks before the experiment, with free access to food. T2DM group rats were fed high fat diet (#MD12033; MediScience Diets Co., Ltd., Yangzhou, China); The Control grou fed all animals with normal diet for 6 months. T2DM rat models were induced by high-fat diet combined with low-dose streptozotocin (Aladdin Co., Ltd., Beijing, China). After fasting for 12 h, rats were intraperitoneally injected with STZ 40 mg/kg [13]. Blood glucose level was measured in tail vein blood 72 h later. If the blood glucose level was ≥ 16.7 mmol/l for 3 consecutive days, the diabetic rat model was considered to be successfully established [14]. Six months after T2DM induction, Euthanasia was carried out by administering an intraperitoneal injection of pentobarbital euthanasia solution (Euthasol®) at a dosage of 100 mg/kg. After the gastrocnemius muscle was cut and weighed, it was divided into three parts: one part was placed in 4% paraformaldehyde solution for hematoxylin-eosin(H&E) staining; The other part was placed in the − 80 refrigerator for qRT-PCR detection; The last part was loaded into liquid nitrogen for transcriptomic detection. The animal study was reviewed and approved by Ethics Committee for Laboratory Animal Welfare of Qingdao University (No.202301SD60202311100). The study is reported in accordance with ARRIVE guidelines.

### Histological evaluation

This is consistent with our previous experimental process [13]. The gastrocnemius muscle were fixed ina neutral solution of paraformaldehyde for 24 h, then paraffin embedding and securing were performed. Conventional H&E staining was observed on automatic scanners (3DHISTECH P250 FLASH, Beijing, China) and images were retained.

### qRT-PCR analysis

Total RNA was extracted from gastrocnemius muscle of T2DM and normal rats by Trizol method [15]. The RNA concentration was detected, cDNA was synthesized by reverse transcription according to the instructions of reverse transcription kit, and qRT-PCR was performed using SYBR Green kit (Q711-02, azyme, Nanjing, China). PCR reaction system 10µL. The specificity of reaction products was verified by the melting curve, and quantitative fluorescence analysis was performed using a real-time fluorescence quantifier (Thermo Fisher Scientific, Shanghai, China). The experiments were repeated 3 times. After the target gene amplification results were corrected by internal reference, the target gene amplification results of the blank group were used as control, and the mRNA expression levels of different samples were compared by 2^−△△CT^. Primer sequences are shown in Table 1.

**Table 1:** Sequencing results of rat gastrocnemius muscle specimen

Samples	Raw reads/bp	Clean reads/bp	Q30/%	GC content/%	Total mapped reads(%)	Uniquely mapped reads(%)
Control group-1	38,025,888	38,025,888	97.20	49	32,350,080(92.65%)	29,786,709(85.31%)
Control group-2	42,410,232	42,410,232	97.46	48	35,387,719(92.86%)	32,419,133(85.07%)
Control group-3	41,435,246	41,435,246	97.15	49	34,609,393(92.20%)	32,035,933(85.34%)
T2DM group-1	42,663,550	42,663,550	96.64	49	34,408,518(89.12%)	31,754,891(82.25%)
T2DM group-2	43,999,056	43,999,056	97.27	51	33,406,661(89.11%)	30,747,882(82.01%)
T2DM group-3	42,771,350	42,771,350	96.62	49	33,853,846(88.78%)	31,428,083(82.42%)

### Transcriptomic analysis

Total RNA was isolated and purified using Trizol reagent (Invitrogen, Carlsbad, CA, USA) following the manufacturer’s procedure. The RNA amount and purity of each sample was quantified using NanoDrop ND-1000 (NanoDrop, Wilmington, DE, USA). After quality control, libraries were constructed as previously described [16]. mRNA-seq were performed on the Hiseq4000 platform (Illumina, CA, USA) with 150 strategy. Lc-Bio Technologies (Hangzhou, China) was responsible for the above sequencings. The DEGs were selected with fold change>2 or fold change<0.5 and with parametric F-test comparing nested linear models (adjusted p-value<0.05) by R package edgeR (https://bioconductor.org/packages/release/bioc/html/edgeR.html).

### Statistical analysis

SPSS 25.0 statistical software (IBM, Armonk, NY, USA) was used for data analysis and processing. Student’s t-test was used to perform statistical comparisons between qRT-PCR data. Figures were plotted using GraphPad Prism 8.0 (La Jolla, CA) with mean ± standard error of the mean (SEM). P value < 0.05 indicated statistical significance.

## Results

### Histologic analysis

**Fig. 1:**
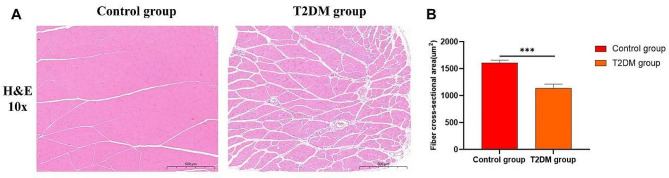
(**A**) Representative images of Hematoxylin and eosin (H&E) staining(10X). (**B**) Fiber cross-sectional area. ***p < 0.001 versus Control group. The values are presented as means, with the error bars depicting the standard deviation

The gastrocnemius tissue of normal rats was orderly, regular in shape, uniform in size, and the nucleus was visible around the muscle fibers. There was no obvious atrophy, edema or rupture of muscle cells. In diabetic rats, the space of gastrocnemius cells increased significantly, and the muscle fibers showed obvious atrophy and inflammatory infiltration **(**Figure 1A**)**. At the same time, the fiber cross-sectional area of gastrocnemius muscle was significantly reduced in T2DM rats compared with control rats (Fig. 1B).

### Filtering and quality assessment of transcriptome sequencing data

**Fig. 2:**
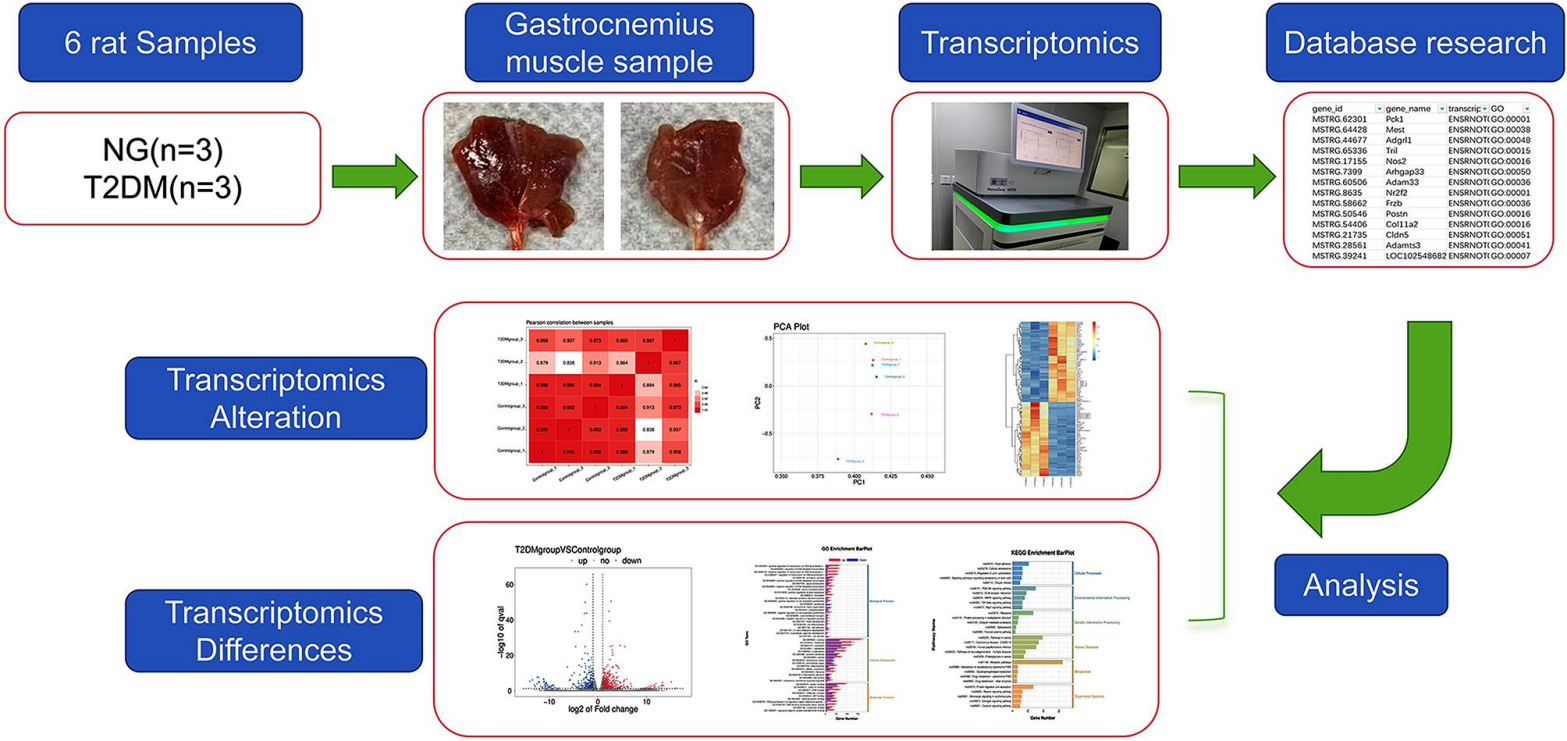
Study design and transcriptomics analysis of the gastrocnemius muscle in diabetic rats. Overview of the cohort (including 3 normal samples and 3 type 2 diabetes samples) and study design (including RNA extraction, library construction, bioinformatics analysis of RNA-seq, and screening of DEGs)

Transcriptomic analysis and study design of gastrocnemius muscle in T2DM rats (Figure 2). After quality control filtering of the original data obtained by transcriptomics, the average value of sample Q30 is 97.06%, and the average GC content is about 49.17%. The quality of the library meets the requirements of subsequent analysis. The results of Unigenes showed that the comparison rates of all samples ranged from 88.78 to 92.86%, and the unique comparison rates ranged from 82.01 to 85.34%. The transcriptome data meets the quality control requirements and can be analyzed later (Table 2).

**Table 2:** Basic information list of 6 DEGs related to apoptosis

Gene name	Gene id	FC	log2(FC)	*p*	Regulation
Map3k14	ENSRNOG00000003278	3.45	1.79	0.00	up
Atf4	ENSRNOG00000017801	2.21	1.14	0.00	up
Pik3r1	ENSRNOG00000018903	2.59	1.37	0.00	up
Il3ra	ENSRNOG00000001325	5.39	2.43	0.00	up
Gadd45b	ENSRNOG00000019822	0.49	-1.02	0.00	down
Bid	ENSRNOG00000012439	2.78	1.47	0.00	up

### DEGs analysis

**Fig. 3:**
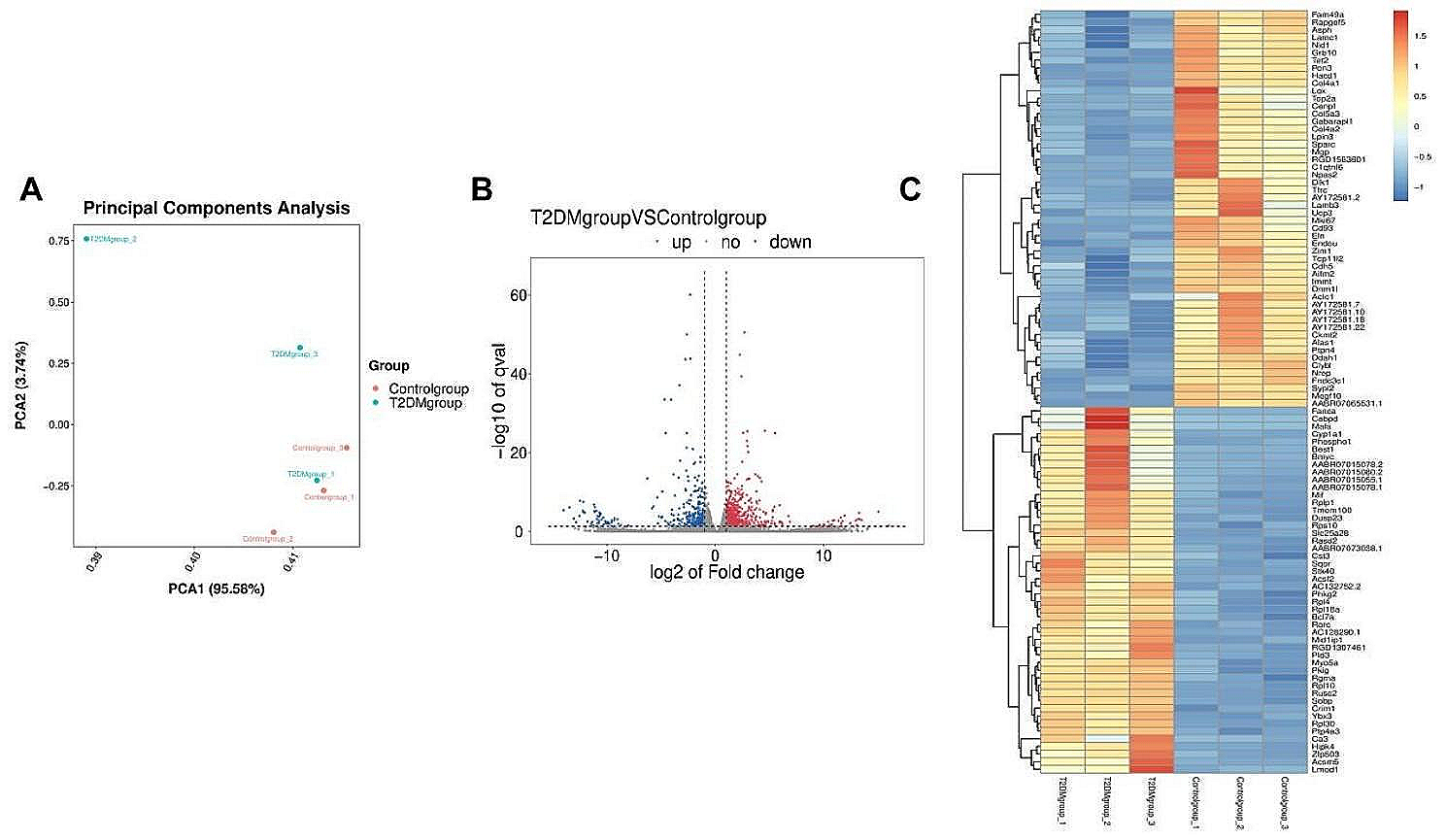
(**A**)Principal component analysis (PCA) analysis. (**B**) Volcano plot of DEGs between two comparison groups. Each dot represents one gene red dots represent the significantly upregulated genes, and blue dots represent the significantly downregulated genes gray dots represent no significant DEGs. (**C**) Heatmap of annotated genes with increasing and decreasing trend. Each column represents a sample, and each gene is visualized in a row red indicates a high abundance, and blue indicates a relatively low abundance of genes

Transcriptomic analysis showed that there were 1016 DEGs between the gastrocnemius of T2DM and normal rats, among which 665 DEGs were up-regulated and 351 DEGs were down-regulated (Additional file 1). The basic information of 6 DEGs related to apoptosis was shown in Table 3. principal component analysis (PCA) analysis was performed on each sample according to the gene expression level. The closer the distance, the higher the similarity between the samples. PCA analysis results showed that T2DM and normal rat gastrocnemius samples were significantly separated, indicating certain differences in the composition of gene expression between the two groups of samples (Fig. 3A). The volcano diagram (Fig. 3B) shows the expression of DEGs directly. Cluster analysis was performed on the two groups of data to obtain the differential gene cluster heat map (Fig. 3C), where the horizontal coordinate represents different samples and the vertical coordinate represents different DEGs.

**Table 3:** Primer information of RT-PCR gene

Gene	Sequence (5’-3’)
GAPDH	Gapdh-F: CTGCCTTCTCTTGTGACAAAGTG
	Gapdh-R: TTGATGACCAGCTTCCCATTCTC
Map3k14	Map3k14-F: GGCTGCTTAGGACTGACTCATTG
	Map3k14-R: GCAAATATAGGGACGGACGGTATC
Atf4	Atf4-F: ATAGAAGAGGTCCGTAAGGCAAGG
	Atf4-R: CAGCAAACACAGCATCACAAGAC
Pik3r1	Pik3r1-F: GCAGGTCTCGTAATGTCGTTCTTG
	Pik3r1-R: TCGCTGACTGACTCTTCCTCTTG
Il3ra	Il3ra-F: GCTGCTGCTGTCGGTTACTATG
	Il3ra-R: GTCGATGCTTAGGTTCCTGATGG
Gadd45b	Gadd45b-F: GAGAGCAGAGGCAATAACCAGTG
	Gadd45b-R: TTTTAGGGGACAGCAACTCAACAG
Bid	Bid-F: GAAGCCAGGTCCCAGAGAACAG
	Bid-R: TCAGCCCTTCAGGTACACTCAAG

NIK, NF-kB-inducing kinase; Gadd45, growth arrest and DNA damage-inducible beta; ATF4, Activating transcription factor 4;Pik3r1, Phosphoinositide-3-kinase regulatory subunit 1; Il3ra, Interleukin 3 receptor subunit alpha; Bid, BH3 interacting domain death agonist;

### GO enrichment analysis and KEGG pathway analysis

**Fig. 4:**
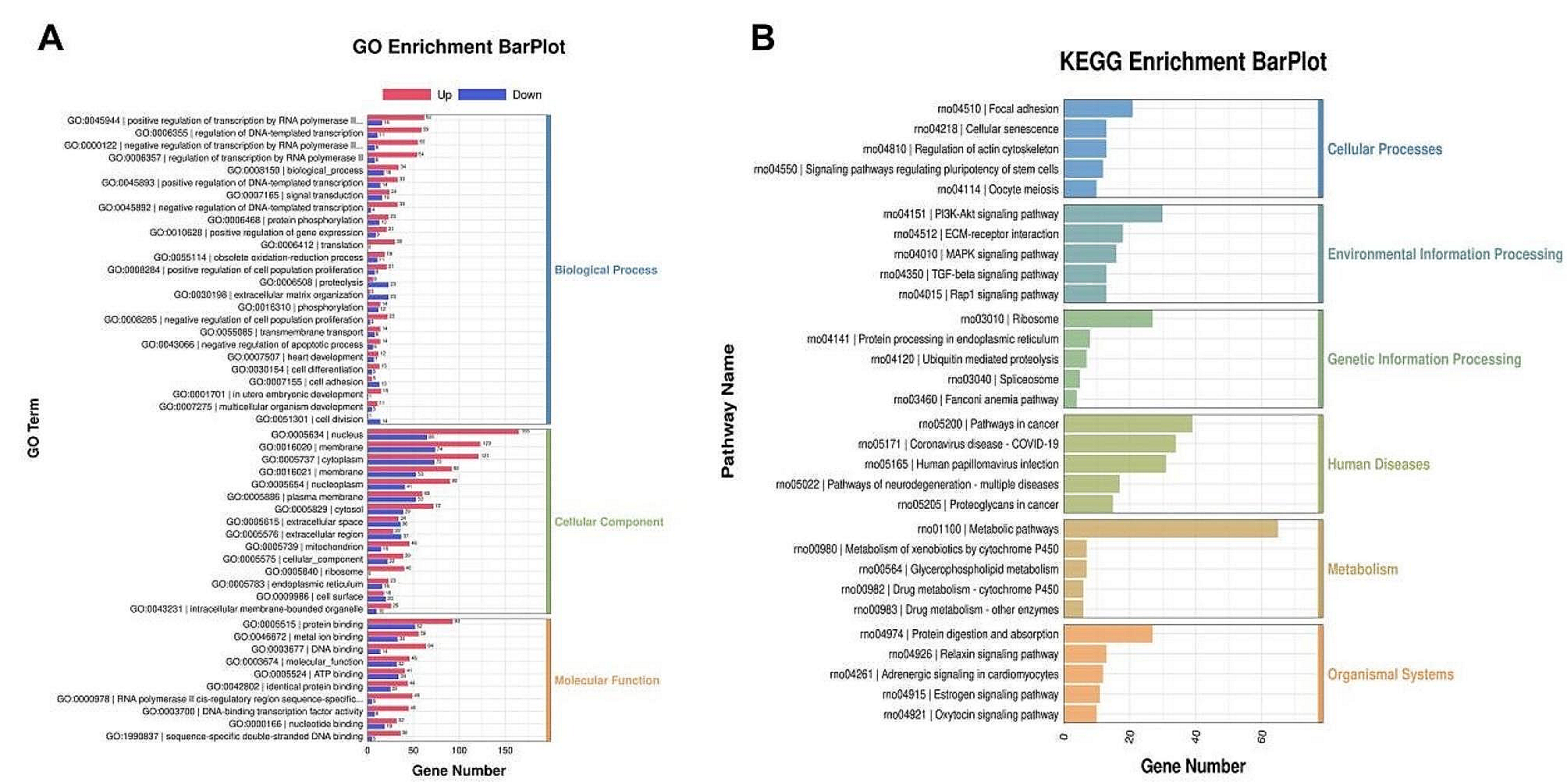
(**A**) According to the principle of Gene ontology (GO) analysis, the biological function of genes is divided into three main parts, namely biological process (BP), cellular component (CC) and molecular function (MF). (**B**) The KEGG functional enrichment analysis in two comparison groups

GO functional enrichment analysis **(**Fig. 4A **and Additional file 2)** showed that the extracellular matrix organization had the most significant enrichment in biological processes, with a total of 26 DEGs. The extracellular matrix with 35 DEGs was the most abundant cell component. The extracellular matrix structural constituent, with 26 DEGs, was the most enriched in molecular functions. The highest number of DEGs enriched in biological processes, cell components and molecular functions were positive regulation of transcription by RNA polymerase II, nucleus and metal ion binding, respectively. There were 78, 230 and 89 DEGs respectively. KEGG pathway enrichment analysis (Fig. 4B and Additional file 3) showed ECM-receptor interaction, PI3K-Akt signaling pathway and TGF-β signaling pathway(*p* < 0.001) showed higher enrichment and differential gene number.

### The expression of DEGs was verified by qRT-PCR

**Fig. 5:**
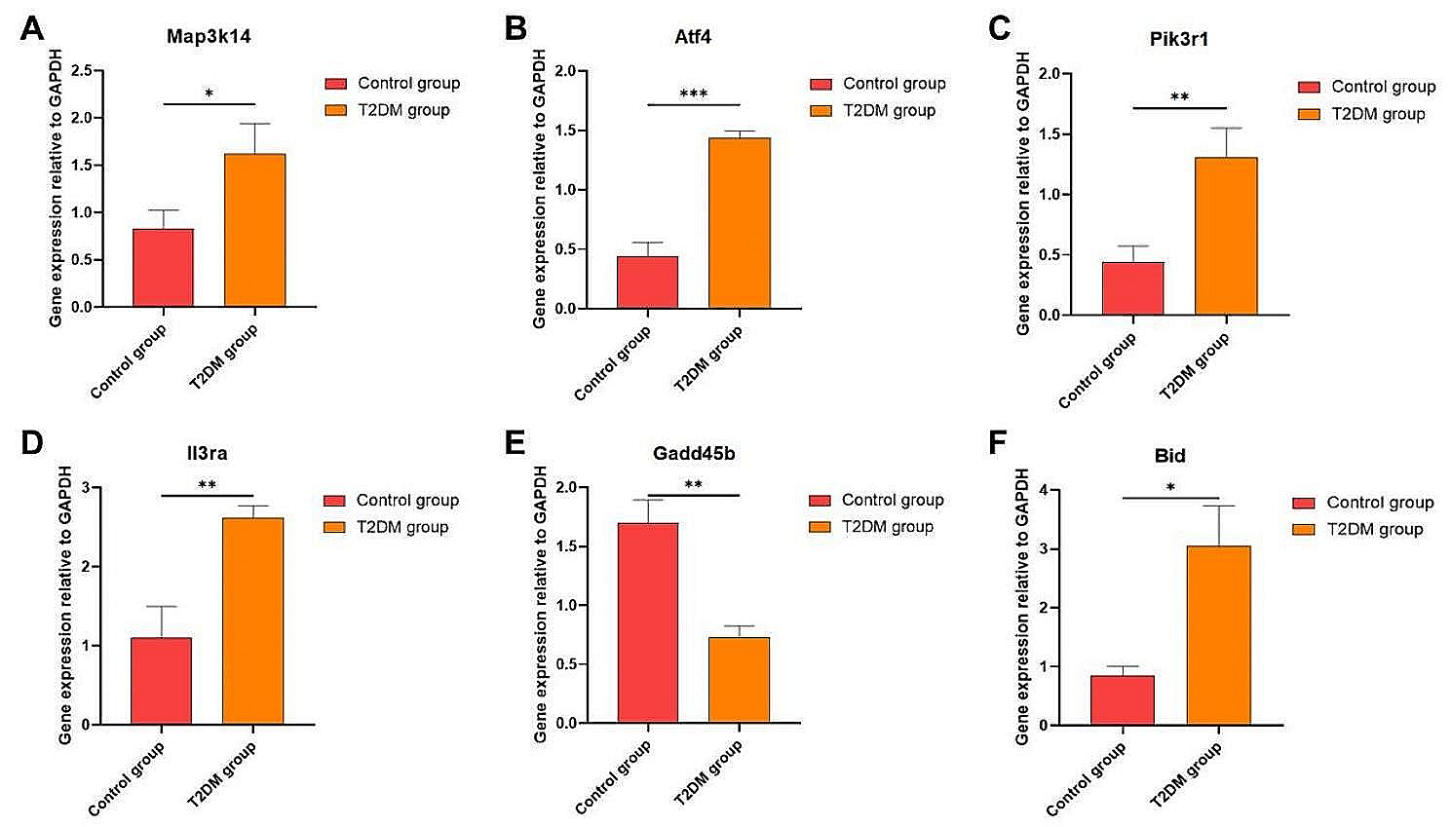
Type 2 diabetes mellitus leads to altered expression of apoptosis-related genes in gastrocnemius muscle(A-F). Error bars depict standard error. *p < 0.05 versus Control group. **p < 0.01 versus Control group. ***p < 0.001 versus Control group. The values are presented as means, with the error bars depicting the standard deviation

Based on the transcriptomic results, in order to further verify and clarify the gene expression differences between T2DM group and Control group, 6 genes related to apoptosis were screened for qRT-PCR verification. qRT-PCR results **(**Fig. 5**)** showed that the acquired fold change of Map3k14, Atf4, Pik3r1, Il3ra, Gadd45b and Bid were 1.95, 3.25, 2.97, 2.38, 0.43 and 3.6, respectively. The fold change of transcriptome sequencing were 3.45, 2.21, 2.59, 5.39, 0.49 and 2.78, respectively. The fold change obtained by qRT-PCR were consistent with those obtained by transcriptome sequencing.

## Discussion

In recent years, the use of molecular biotechnology to find potential biomarkers of diseases has become an important means to explore the pathogenesis of sarcopenia [7–9]. This study was the first to establish the “gene profiles” of the gastrocnemius muscle of T2DM and normal rats by high-throughput sequencing, and 1016 DEGs were found. The enrichment analysis of KEGG pathways involved 299 pathways. ECM-receptor interaction, PI3K-Akt signaling pathway and TGF-β signaling pathway showed higher enrichment and differential gene number. These results may provide new ideas for exploring the pathogenesis of diabetic sarcopenia. In addition, this study used bioinformatics analysis to screen out and verify important genes related to apoptosis. Some of the targeted genes screened in this study have rarely been reported in the regulation of muscle apoptosis, and these genes provide valuable information for further investigation of the pathogenesis of diabetic sarcopenia.

Although the exact mechanisms underlying sarcopenia are far to be unveiled, a growing body of experimental evidence suggests that Nuclear apoptosis may lead to atrophy and sarcopenia. Chronic increased activity weakens apoptosis signaling, which may reduce sarcopenia [17, 18]. Targeting apoptosis during aging may provide a new and effective therapeutic tool for combating sarcopenia [19]. Meanwhile, apoptosis is associated with acute disuse muscular atrophy, and Caspase-3-dependent pathway plays an important role in this process [20]. Based on the above studies, we believe that further identifying the most relevant apoptotic pathways to target and determining the best time to intervene may be an effective measure to treat sarcopenia. Based on the above relevant studies, we screened the differential genes involved in apoptosis, which can be used as an entry point to explore the mechanism of diabetic sarcopenia. The mRNAs with 6 differentially expressed genes were detected by qRT-PCR. The results showed that all the 6 genes showed different degrees of differentially expressed genes. The results of qRT-PCR were consistent with those of transcriptome sequencing, which confirmed the accuracy and reliability of sequencing results.

We found that NF-kB-inducing kinase (NIK) may be a novel, previously unrecognized biomarker of T2DM-induced skeletal muscle atrophy. The dysfunction of NIK(also known as Map3k14) is involved in the development of a variety of autoimmune diseases and inflammation [21]. Previous literature found that overexpression of NIK induced a 30% decrease in the mean cross-sectional fiber area of skeletal muscle, which was related to increased mRNA expression of growth arrest and DNA damage-inducible beta (Gadd45), biomarkers of skeletal muscle atrophy [21]. In addition, elevated NIK levels also induce skeletal muscle insulin resistance [22, 23] and promote muscle atrophy associated with cancer cachexia [24]. In relation to liver glycogen production, the absence of NIK in the liver can prevent liver steatosis induced by high fat diet and the reduction of liver glycogen production [25]. Although more and more studies have confirmed that overexpression of NIK is associated with muscle atrophy, our study is the first to report NIK expression in gastrocnemius in diabetic conditions. However, in the future, we still need to verify the correlation between NIK overexpression and atrophic gene expression in high-glucose environment through more detailed cell experiments.

As a growth retarding and DNA damage inducing protein, Gadd45 plays a critical regulatory role in various cellular functions, such as DNA repair, cell cycle regulation and aging, and genotoxic stress response. Activation or overexpression of Gadd45 has been reported to inhibit cell growth and induce cell death [26]. Similarly, our results also confirmed that the expression of Gadd45 in gastrocnemius of T2DM rats was significantly increased. Previous literature has also confirmed that Gadd45 is involved in various cellular processes, including maintenance of genome integrity, growth arrest, apoptosis [26], senescence, and signal transduction [27]. Disuse muscle atrophy in elderly rats is closely related to the overexpression of Gadd45 [28]. However, the specific mechanism of how the increased expression of Gadd45 in gastrocnemius muscle leads to sarcopenia through apoptosis in T2DM rats still needs to be further studied.

Previous studies have found that Activating transcription factor 4 (ATF4) can be correlated with muscle atrophy [29–31]. Studies have found that ATF4 controls endoplasmic reticulum stress, oxidative stress, mitochondrial autophagy, inflammatory response, iron death, synaptic plasticity and other processes, and plays various roles in various pathophysiological processes [31]. Under various stress conditions such as aging, fasting or limb immobilization, the up-regulation of ATF4 expression in skeletal muscle fibers can cause atrophy of muscle fibers [32]. By activating specific genes in muscle fibers, ATF4 contributes to the expression of many mrnas, including those that encode the mediator of muscle fiber atrophy, such as GADD45. ATF4 is a mediator of skeletal muscle aging, and ATF4 gene knockout mice showed significant protective effects in terms of muscle mass as they aged [32]. Overall, our data suggest that ATF4 expression is significantly increased in gastrecnemius of T2DM rats, and ATF4 may be an important mediator of skeletal muscle atrophy in diabetic rats.

BH3 interacting domain death agonist(BID)can act as a BAX-like effector of apoptosis. BID can also mediate mitochondrial permeabilization by itself, resulting in release of cytochrome c and mitochondrial DNA, caspase activation and apoptosis [33, 34]. Phosphoinositide-3-kinase regulatory subunit 1 (Pik3r1) is involved in the pathogenesis of chronic glucocorticoid-induced skeletal muscle atrophy, and Pik3r1 knockout mice have larger muscle tube diameter [35]. In addition, MicroRNA-106a-5p inhibits C2C12 Myogenesis by targeting PIK3R1 [36]. Pik3r1 and the downstream effector AKT are abnormally expressed in apoptosis progression. After activation, AKT can regulate many downstream target proteins, such as apoptosis, survival and phosphorylation of mTOR protein. Therefore, Pik3r1 regulates transcription and protein synthesis, and plays an important role in cell growth and apoptosis [35]. Although we found an increased expression of Il3ra in the gastrocnemius muscle of T2DM rats, there is currently no clear correlation between Il3ra and muscular dystrophy, and our results may provide new insights into the pathogenesis of diabetic sarcopenia.

At present, research on sarcopenia mainly focuses on patients with long-term bed rest and cachexia, and relatively few studies have been conducted on animals or humans with T2DM. In this study, high-throughput sequencing technology was used to obtain transcriptome information of gastrocnemius muscle of T2DM and normal rats for the first time, and the results of this study may provide new ideas for exploring the pathogenesis of diabetic sarcopenia. Most importantly, our dataset provides a wealth of differentially expressed genes, each of which provides a foundation for future research. The data could be useful to future researchers, giving researchers access to a large subset of genes. However, our study still has some limitations. First, the exact function of these differentially expressed genes and their role in sarcopenia need to be further studied in order to provide a basis for the treatment of diabetic sarcopenia. Secondly, this study is limited to the transcriptomic level, and other technical methods such as proteomics and metabolomics need to be further supplemented and improved, which can provide multidimensional verification of the molecular mechanism of this model. Finally, in this study, only qRT-PCR was used to verify the expression of part of DEGs without clarifying the pathogenic mechanism, which will be further studied in the future.

## Conclusions

Transcriptome sequencing was performed on gastrocnemius muscle of T2DM and normal rats. KEGG pathway enrichment analysis showed that ECM-receptor interaction, PI3K-Akt signaling pathway and TGF-β signaling pathway had higher enrichment degree and number of differential genes. A total of 1016 DEGs were screened between the two groups, and the related genes that may affect apoptosis were screened and verified. The results showed that Map3k14, Atf4, Pik3r1, Il3ra, Gadd45b and Bid had significant changes in gastrocnemius muscle of T2DM rats, suggesting that these genes may be involved in the pathogenesis of diabetic sarcopenia. These results may lay a foundation for studying the molecular mechanism of diabetic sarcopenia.

### Electronic supplementary material

Below is the link to the electronic supplementary material.

Supplementary Material 1

Supplementary Material 2

Supplementary Material 3

## Data Availability

The datasets generated and/or analyzed during the current study are available in the Gene Expression Omnibus (GEO) repository, accession number GSE268372 https://www.ncbi.nlm.nih.gov/geo/.

## Abbreviations

T2DM: Type 2 diabetes mellitus
DEGs: Differentially expressed genes
GO: gene ontology
KEGG: Kyoto encyclopedia of genes and gnomes
NIK: NF-kB-inducing kinase
Gadd45: Growth arrest and DNA damage-inducible beta
ATF4: Activating transcription factor4
Pik3r1: Phosphoinositide-3-kinase regulatory subunit 1
Il3ra: Interleukin 3 receptor subunit alpha
Bid: BH3 interacting domain death agonist
